# Safety and Efficacy of Vadadustat Versus Darbepoetin Alfa for Chronic Kidney Disease–Related Anemia in Patients Receiving Dialysis by Baseline Erythropoiesis‐Stimulating Agent Dose

**DOI:** 10.1111/hdi.70034

**Published:** 2025-12-11

**Authors:** Alan Jardine, Steven K. Burke, Wenli Luo, Todd Minga, Mark J. Sarnak, Wolfgang C. Winkelmayer, Rajiv Agarwal, Glenn M. Chertow, Kai‐Uwe Eckardt, Mark J. Koury

**Affiliations:** ^1^ Institute of Cardiovascular and Medical Sciences University of Glasgow Glasgow UK; ^2^ Akebia Therapeutics, Inc. Cambridge Massachusetts USA; ^3^ Division of Nephrology Tufts Medical Center Boston Massachusetts USA; ^4^ Section of Nephrology, Department of Medicine Baylor College of Medicine Houston Texas USA; ^5^ Department of Medicine, and Richard L. Roudebush Veterans Administration Medical Center Indiana University School of Medicine Indianapolis Indiana USA; ^6^ Departments of Medicine, Epidemiology and Population Health, and Health Policy Stanford University School of Medicine Palo Alto California USA; ^7^ Department of Nephrology and Medical Intensive Care Charité–Universitätsmedizin Berlin Berlin Germany; ^8^ Division of Hematology/Oncology Vanderbilt University Medical Center Nashville Tennesse USA

**Keywords:** anemia, CKD, dialysis, ESA, HIF‐PHI

## Abstract

**Introduction:**

Erythropoiesis‐stimulating agents (ESAs) and iron supplementation are standard treatments for chronic kidney disease (CKD)–related anemia. Targeting higher hemoglobin values in CKD increases cardiovascular risk. Whether the increased risk is from higher ESA doses or higher hemoglobin levels is uncertain, but alternative therapies are sought for patients requiring high ESA doses. Phase 3 INNO_2_VATE trials in patients with dialysis‐dependent CKD (DD‐CKD) demonstrated vadadustat's noninferiority compared with darbepoetin alfa. To determine vadadustat's potential to treat anemia, including in patients requiring high ESA doses, its safety and efficacy were compared with those of darbepoetin alfa across prespecified baseline ESA dose subgroups in the prevalent DD‐CKD INNO_2_VATE trial.

**Methods:**

We compared the safety and efficacy of vadadustat versus darbepoetin alfa across prespecified baseline ESA dose subgroups (low [≤ 90 U/kg/week], intermediate [> 90 and < 300 U/kg/week], or high [≥ 300 U/kg/week]) in the INNO_2_VATE prevalent trial. The primary safety endpoint was time to first adjudicated major adverse cardiovascular event (MACE). Primary and secondary efficacy endpoints were mean hemoglobin level change from baseline at weeks 24–36 and weeks 40–52, respectively.

**Findings:**

Compared with darbepoetin alfa, first MACE hazard ratios for vadadustat were 0.99 (95% CI, 0.81–1.23), 0.93 (95% CI, 0.74–1.18), and 0.62 (95% CI, 0.34–1.14) for low, intermediate, and high baseline ESA dose subgroups, respectively (interaction *p =* 0.92). Vadadustat was noninferior to darbepoetin alfa in hemoglobin change from baseline to primary evaluation period, with mean differences (vadadustat–darbepoetin alfa) of −0.10 g/dL (95% CI, −0.19 to −0.02), −0.20 g/dL (95% CI, −0.30 to −0.09), and −0.39 g/dL (95% CI, −0.67 to −0.11) for low, intermediate, and high ESA dose subgroups, respectively.

**Discussion:**

Comparing safety and efficacy by baseline ESA dose among patients with CKD on maintenance dialysis, vadadustat was noninferior to darbepoetin alfa for all ESA dose subgroups, including patients with high baseline ESA requirements.

## 1 Introduction

Anemia complicating chronic kidney disease (CKD) is associated with increased morbidity, mortality, and health care resource utilization [1]. Anemia prevalence increases with advanced CKD, primarily due to inflammation‐related processes: impaired erythropoietin (EPO) production [2, 3, 4] and restricted iron availability for erythropoiesis [4, 5]. Inflammation in CKD transforms interstitial renal cortical EPO‐producing fibroblasts into EPO‐nonproducing myofibroblasts [6]. Inflammation suppresses erythropoietic progenitor and precursor cells via pro‐apoptotic and inhibitory cytokines [7]. Inflammation indirectly suppresses erythropoiesis via the cytokine interleukin‐6, which increases liver production of hepcidin, the down‐regulator of ferroportin [8].

Standard treatments for CKD–related anemia include (1) recombinant human EPO or longer‐acting derivatives such as darbepoetin alfa (hereafter “darbepoetin”) or methoxy polyethylene glycol–epoetin beta, collectively termed erythropoiesis‐stimulating agents (ESAs); and (2) intravenous or oral iron supplementation to accommodate reduced intestinal iron absorption and increased iron sequestration [7, 9, 10]. However, responses to treatment vary significantly. Some patients cannot achieve and/or maintain target hemoglobin concentrations despite escalating ESA doses and large intravenous iron doses, especially in dialysis‐dependent (DD)‐CKD [11]. Safety concerns with escalating ESA doses include an increased risk for death, cardiovascular events, and cancer progression [12, 13, 14]. Targeting higher hemoglobin values in CKD patients increases cardiovascular risk [12, 13, 14], which may be related to higher ESA doses and/or higher hemoglobin. Excessive intravenous iron increases the risk for iron deposition in organs, infections, and atherosclerosis [15, 16, 17, 18]. Therefore, alternative therapies are sought for patients requiring high ESA and/or iron doses.

Vadadustat, a hypoxia‐inducible factor prolyl hydroxylase inhibitor (HIF‐PHI) that stimulates erythropoiesis [19, 20, 21, 22], is approved for treating anemia in DD‐CKD in many countries, including the United States. Vadadustat increases endogenous EPO in plasma above baseline without producing larger increases associated with ESAs [23]. Vadadustat improves iron availability by decreasing serum hepcidin while increasing iron and total iron‐binding capacity (TIBC) [24], resulting in increased red blood cells (RBCs), mean corpuscular volume, and mean corpuscular hemoglobin. By these mechanisms, vadadustat may safely ameliorate anemia in patients with DD‐CKD who require high ESA doses.

To determine vadadustat's potential to treat anemia in patients requiring high ESA doses, its safety and efficacy were compared with darbepoetin across prespecified baseline ESA dose subgroups in the prevalent DD‐CKD INNO_2_VATE trial (NCT02892149). Patients on maintenance dialysis were stratified by ESA doses at trial baseline into low (≤ 90 U/kg/week), intermediate (> 90 and < 300 U/kg/week), or high (≥ 300 U/kg/week) subgroups. In the prevalent INNO_2_VATE trial, vadadustat met the prespecified primary safety endpoint of time to major adverse cardiovascular events (MACE) and was noninferior to darbepoetin [19]. Vadadustat also met the primary efficacy endpoint of mean change in hemoglobin from baseline during the primary evaluation period (PEP) by elevating and maintaining hemoglobin in patients already receiving, or starting, maintenance dialysis [19]. Understanding vadadustat safety and efficacy in ESA dose subgroups will help optimize vadadustat usage, irrespective of responses to standard therapy.

## 2 Materials and Methods

### 2.1 Study Design

The prevalent DD‐CKD INNO_2_VATE trial, a phase 3, global, open‐label, sponsor‐blind, parallel‐group, active‐controlled, noninferiority trial, compared the safety and efficacy of vadadustat with darbepoetin in patients with DD‐CKD [19]. Rationale, methods, and primary results have been reported [19, 25]. The trial was performed in compliance with the International Conference on Harmonization, Good Clinical Practice guidelines, local regulatory requirements and laws, and the Declaration of Helsinki. Institutional review board approval was obtained at participating sites. All patients provided written informed consent before enrollment. Analyses reported herein were conducted by prespecified ESA baseline dose subgroups.

### 2.2 Study Population

Patients were aged ≥ 18 years, treated with hemodialysis or peritoneal dialysis for ≥ 12 weeks before screening, receiving ESA therapy at enrollment, and had baseline hemoglobin of 8–11 g/dL (US) or 9–12 g/dL (non‐US), serum ferritin ≥ 100 ng/mL, and transferrin saturation ≥ 20%. Patients were excluded for RBC transfusion within 8 weeks before randomization, anemia secondary to causes other than CKD, uncontrolled hypertension, or recent cardiovascular event [19].

### 2.3 Study Procedures

Patients were randomized 1:1 to vadadustat or darbepoetin, stratified by geographic region (USA/Europe/other regions), New York Heart Association Functional Classification (0/I vs. II/III), and hemoglobin concentration (< 10 vs. ≥ 10 g/dL). The trial had 4 periods: (1) conversion (weeks 0–23); (2) maintenance (weeks 24–52), which included primary (weeks 24–36) and secondary (weeks 40–52) evaluation periods; (3) long‐term treatment (week 53 to end of treatment [182 weeks]); and (4) post‐treatment 4‐week safety follow‐up [19, 25].

Initial vadadustat dose for all patients was not based on baseline ESA dose, but was fixed at 300 mg orally once daily, with doses of 150, 300, 450, and 600 mg available for adjustments. Maximum vadadustat daily dose was 600 mg. Darbepoetin was administered intravenously for patients on maintenance hemodialysis and subcutaneously for patients receiving peritoneal dialysis at the site facility or at home according to the investigator's determination and local practice. Initial trial dose of darbepoetin was based on prerandomization dose for patients already receiving darbepoetin, or on local product label if receiving another ESA before randomization.

Trial doses were adjusted according to the investigator's discretion, incorporating protocol‐based guidance, and considering the patient's clinical condition and hemoglobin trajectory [19, 25]. Hemoglobin concentrations were measured every 2 weeks for weeks 0–12 and every 4 weeks for weeks 12–52, and doses were adjusted accordingly. Investigators aimed to maintain hemoglobin within geography‐specific target ranges (10–11 g/dL in the USA; 10–12 g/dL in other countries). Iron supplementation was encouraged to maintain serum ferritin ≥ 100 ng/mL or transferrin saturation ≥ 20%.

Starting at 6 weeks, patients could receive rescue therapy for worsening anemia symptoms with hemoglobin < 9.5 g/dL. Rescue therapy was defined as receiving an ESA or RBC transfusion in the vadadustat group, and receiving another ESA, a two‐fold or greater increase over the previous darbepoetin dose, or RBC transfusion in the darbepoetin group.

### 2.4 Endpoints

Safety and efficacy were evaluated in prespecified subgroups of patients categorized by their baseline intravenous epoetin alfa or epoetin alfa–equivalent doses (low [≤ 90 U/kg/week], intermediate [> 90 and < 300 U/kg/week], or high [≥ 300 U/kg/week; a threshold for characterizing ESA resistance]) [26]. Conversions for other ESAs to intravenous epoetin alfa equivalents (U/kg/week) were: darbepoetin, 1:200 [27, 28]; methoxy polyethylene glycol–epoetin beta, 1:220 [29]; and subcutaneous epoetin alfa, 1:1.25 [30, 31]. The prespecified primary safety endpoint was the first occurrence of an adjudicated MACE (all‐cause mortality, nonfatal myocardial infarction, or nonfatal stroke) using time‐to‐event analysis. Additional related endpoints included all‐cause mortality, MACE plus hospitalizations for heart failure or thromboembolic events, the first occurrence of cardiovascular MACE (cardiovascular mortality, nonfatal myocardial infarction, or nonfatal stroke), and cardiovascular death [19]. We also assessed treatment‐emergent serious adverse events of special interest.

The prespecified primary efficacy endpoint was mean change in hemoglobin from baseline to PEP (weeks 24–36); the prespecified secondary endpoint was mean change in hemoglobin from baseline to secondary evaluation period (SEP; weeks 40–52) [19]. We also assessed for each baseline ESA dose subgroup: (1) number of patients with average hemoglobin within geography‐specific target range during the PEP and SEP; (2) incidence of RBC transfusions and ESA rescues; (3) vadadustat and darbepoetin dose over the PEP and SEP; (4) iron‐related and inflammatory parameters (serum TIBC, hepcidin, ferritin, iron, transferrin saturation, and C‐reactive protein) [25]; and (5) serum EPO/mL, reticulocytes/μL, RBCs/μL, and RBC indices (mean corpuscular volume, mean corpuscular hemoglobin, and RBC distribution width). Post hoc analyses were performed for interactions between treatment and ESA baseline dose for both MACE and hemoglobin change from baseline.

## 3 Statistics

Analysis of time to first MACE was based on a Cox regression model and included covariates of baseline hemoglobin, randomization strata of region (USA, Europe, other regions), New York Heart Association class (0 or I/II or III), sex, age (> 65 years/≤ 65 years), race (White/non‐White), pre‐existing cardiovascular disease (yes/no), and diabetes mellitus (yes/no). We calculated hazard ratios (vadadustat/darbepoetin) and 95% CIs from model parameter estimates and standard errors, respectively. For the primary efficacy endpoint, noninferiority of vadadustat was achieved if the lower bound of the 95% CI was ≥ −0.75, applied to the difference in mean change (vadadustat–darbepoetin).

In efficacy analyses, we used multiple imputation with analysis of covariance, with covariates of treatment, baseline hemoglobin, and stratification factors (region, New York Heart Association class). Missing data were addressed through multiple imputations using a fully conditional specification approach. No multiplicity adjustments were applied for MACE or efficacy analyses in the subgroups, although in the overall population sequential testing was employed for primary and secondary efficacy endpoints and MACE‐related endpoints to control type I error. Descriptive statistics were captured for the incidence of RBC transfusions and ESA rescue by baseline ESA dose subgroup. We compared results for iron‐related parameters, inflammatory markers, serum endogenous EPO, reticulocytes, and RBC indices using least‐squares (LS) mean change from baseline and differences (vadadustat–darbepoetin; 95% CI) during the PEP and SEP. For the efficacy analysis, we further tested the interaction between baseline ESA dose and treatment group in the analysis of covariance model.

## 4 Results

### 4.1 Baseline Characteristics

In the prevalent DD‐CKD INNO_2_VATE trial, 3554 patients underwent randomization (vadadustat, 1777; darbepoetin, 1777). Baseline characteristics were similar across treatment groups and baseline ESA dose subgroups (Table 1), except for race and body mass index. The proportion of Black patients was directly related to baseline ESA dose and mean body mass index was inversely related. Additionally, > 80% of patients in the high baseline ESA dose subgroup were from the USA, whereas ~60% were from the USA in the low and intermediate baseline ESA dose subgroups. Mean hemoglobin concentrations were slightly lower in the high baseline ESA dose subgroup compared with the low and intermediate dose subgroups.

**TABLE 1:** Selected baseline characteristics of patients in the prevalent DD‐CKD INNO_2_VATE trial by baseline ESA dose (randomized population).

Characteristic	≤ 90 U/kg/week		> 90 and < 300 U/kg/week		≥ 300 U/kg/week	
	Vadadustat (*n* = 916)	Darbepoetin alfa (*n* = 968)	Vadadustat (*n* = 724)	Darbepoetin alfa (*n* = 693)	Vadadustat (*n* = 102)	Darbepoetin alfa (*n* = 98)
Mean age, years (SD)	58.5 (13.6)	59.7 (13.2)	57.5 (14.3)	56.7 (14.8)	57.1 (13.4)	57.1 (12.6)
Sex, male, *n* (%)	517 (56.4)	569 (58.8)	396 (54.7)	383 (55.3)	56 (54.9)	46 (46.9)
Racial or ethnic group, *n* (%)						
White	626 (68.3)	635 (65.6)	429 (59.3)	398 (57.4)	58 (56.9)	48 (49.0)
Asian	26 (2.8)	41 (4.2)	42 (5.8)	43 (6.2)	7 (6.9)	14 (14.3)
Black or African American	203 (22.2)	217 (22.4)	189 (26.1)	194 (28.0)	33 (32.4)	31 (31.6)
Other^a^	61 (6.7)	75 (7.7)	64 (8.8)	58 (8.4)	4 (3.9)	5 (5.1)
Hispanic ethnic group, *n* (%)						
Hispanic/Latino	329 (35.9)	337 (34.8)	289 (39.9)	287 (41.4)	42 (41.2)	38 (38.8)
Not Hispanic/Latino	561 (61.2)	593 (61.3)	411 (56.8)	383 (55.3)	58 (56.9)	59 (60.2)
Not reported/unknown	26 (2.8)	38 (3.9)	24 (3.3)	23 (3.3)	2 (2.0)	1 (1.0)
Region of enrollment, *n* (%)						
USA	564 (61.6)	590 (61.0)	424 (58.6)	406 (58.6)	85 (83.3)	81 (82.7)
Europe	152 (16.6)	186 (19.2)	95 (13.1)	91 (13.1)	2 (2.0)	2 (2.0)
Non‐US/Europe	200 (21.8)	192 (19.8)	205 (28.3)	196 (28.3)	15 (14.7)	15 (15.3)
Mean time since dialysis started, years (SD)	4.0 (3.9)	3.9 (4.0)	4.1 (4.2)	4.0 (4.1)	4.0 (4.2)	4.5 (4.0)
Type of dialysis, *n* (%)						
Hemodialysis	859 (93.8)	903 (93.3)	676 (93.4)	638 (92.1)	88 (86.3)	86 (87.8)
Peritoneal dialysis	57 (6.2)	65 (6.7)	47 (6.5)	55 (7.9)	14 (13.7)	12 (12.2)
Disease history, *n* (%)						
Diabetes mellitus	514 (56.1)	569 (58.8)	389 (53.7)	361 (52.1)	52 (51.0)	58 (59.2)
Cardiovascular disease	454 (49.6)	531 (54.9)	351 (48.5)	344 (49.6)	48 (47.1)	54 (55.1)
New York Heart Association Functional Classification, *n* (%)						
0 (no CHF) or I	782 (85.4)	832 (86.0)	643 (88.8)	612 (88.3)	92 (90.2)	87 (88.8)
II or III	134 (14.6)	136 (14.0)	81 (11.2)	81 (11.7)	10 (9.8)	11 (11.2)
Mean body mass index, kg/m^2^ (SD)	29.8 (7.8)	29.8 (7.3)	27.4 (6.4)	27.3 (6.7)	25.9 (5.8)	25.4 (7.0)
Baseline weight						
Mean (SD)	84.3 (22.8)	83.9 (22.1)	76.5 (19.8)	76.2 (21.2)	72.5 (19.7)	69.4 (19.8)
Median (Q1, Q3)	80.9 (69.0, 96.0)	81.0 (68.5, 96.6)	74.5 (62.5, 86.8)	73.0 (61.0, 85.0)	71.0 (59.0, 84.2)	66.3 (57.0, 77.4)
≤ 60 kg, *n* (%)	101 (11.0)	119 (12.3)	149 (20.6)	146 (21.1)	28 (27.5)	30 (30.6)
> 60 and ≤ 90 kg, *n* (%)	504 (55.0)	516 (53.3)	426 (58.8)	430 (62.0)	54 (52.9)	58 (59.2)
> 90 kg, *n* (%)	311 (34.0)	333 (34.4)	149 (20.6)	117 (16.9)	20 (19.6)	10 (10.2)
Mean hemoglobin concentration, g/dL (SD)	10.3 (0.81)	10.3 (0.77)	10.2 (0.87)	10.2 (0.88)	9.72 (0.91)	9.85 (0.85)
Baseline hemoglobin category, *n* (%)						
< 10.0 g/dL	282 (30.8)	302 (31.2)	262 (36.2)	262 (37.8)	62 (60.8)	49 (50.0)
≥ 10.0 g/dL	634 (69.2)	666 (68.8)	462 (63.8)	431 (62.2)	40 (39.2)	49 (50.0)
Baseline ESA use, *n* (%)						
Epoetin	429 (46.8)	470 (48.6)	445 (61.5)	413 (59.6)	77 (75.5)	70 (71.4)
Darbepoetin alfa	318 (34.7)	346 (35.7)	156 (21.5)	163 (23.5)	8 (7.8)	11 (11.2)
Methoxy polyethylene glycol–epoetin beta	169 (18.4)	152 (15.7)	123 (17.0)	117 (16.9)	17 (16.7)	17 (17.3)
Baseline ESA dose (U/kg/week)^b^						
Mean (SD)	48.4 (22.0)	49.2 (22.5)	156.3 (51.4)	152.6 (50.6)	447.0 (138.2)	443.4 (180.4)
Median (Q1, Q3)	46.5 (30.4, 66.6)	49.4 (31.0, 67.4)	145.1 (114.3, 187.5)	140.4 (111.7, 181.8)	404.0 (337.8, 492.7)	388.5 (337.1, 472.4)
Iron parameters and C‐reactive protein						
Baseline iron use, *n* (%)						
Oral iron only	64 (7.0)	62 (6.4)	46 (6.4)	42 (6.1)	7 (6.9)	8 (8.2)
Intravenous iron only	471 (51.4)	469 (48.5)	381 (52.6)	335 (48.3)	42 (41.2)	42 (42.9)
Oral and intravenous iron	37 (4.0)	49 (5.1)	33 (4.6)	30 (4.3)	12 (11.8)	6 (6.1)
Did not receive iron	344 (37.6)	388 (40.1)	264 (36.5)	286 (41.3)	41 (40.2)	42 (42.9)
TIBC (mean [SD]), μg/dL (250–425 μg/dL^c^)	*n* = 915 211.9 (34.9)	*n* = 968 213.3 (35.4)	*n* = 723 207.3 (36.4)	*n* = 693 210.1 (36.5)	*n* = 101 209.1 (42.5)	*n* = 98 202.7 (41.4)
Hepcidin (mean [SD]), ng/mL (male: 8.6–82.6 ng/mL; female: 6.2–51.5 ng/mL^c^)	*n* = 907 198.8 (138.9)	*n* = 952 194.1 (132.3)	*n* = 715 191.9 (140.9)	*n* = 686 187.5 (139.6)	*n* = 102 173.8 (144.0)	*n* = 96 178.9 (146.1)
Ferritin (mean [SD]), ng/mL (10–380 ng/mL^c^)	*n* = 916 876 (569)	*n* = 968 855 (527)	*n* = 723 824 (563)	*n* = 693 829 (560)	*n* = 102 798 (520)	*n* = 98 832 (496)
Serum iron (mean [SD]), μg/dL (male: 65–175 μg/dL; female: 50–170 μg/dL^c^)	*n* = 916 82.1 (28.3)	*n* = 968 80.6 (27.4)	*n* = 723 77.3 (30.0)	*n* = 693 77.3 (28.0)	*n* = 102 75.9 (33.3)	*n* = 98 75.0 (33.7)
Transferrin saturation (mean [SD]), % (male: 20%–50%; female: 15%–50%^c^)	*n* = 915 39.2 (13.5)	*n* = 968 38.1 (13.1)	*n* = 723 37.2 (13.3)	*n* = 693 37.0 (12.9)	*n* = 101 35.9 (13.7)	*n* = 98 37.5 (15.4)
C‐reactive protein (mean [SD]), mg/L (0.0–4.9 mg/L^c^)	*n* = 904 9.2 (17.9)	*n* = 947 9.4 (17.6)	*n* = 716 11.3 (22.9)	*n* = 680 9.5 (16.3)	*n* = 102 11.6 (16.8)	*n* = 96 13.4 (21.5)

Abbreviations: CHF, congestive heart failure; DD‐CKD, dialysis‐dependent chronic kidney disease; ESA, erythropoiesis‐stimulating agent; Q1, lower quartile; Q3, upper quartile; TIBC, total iron‐binding capacity.

^a^ Includes American Indian or Alaska Native, Native Hawaiian, or other Pacific Islander, multiple or not reported.

^b^ ESA doses were converted to intravenous epoetin equivalent unit per kilogram per week (U/kg/week): darbepoetin alfa to intravenous epoetin was 1:200; methoxy polyethylene glycol–epoetin beta to intravenous epoetin was 1:220; subcutaneous epoetin to intravenous epoetin was 1:1.25.

^c^ Reference ranges [32, 33].

### 4.2 MACE

**FIGURE 1:**
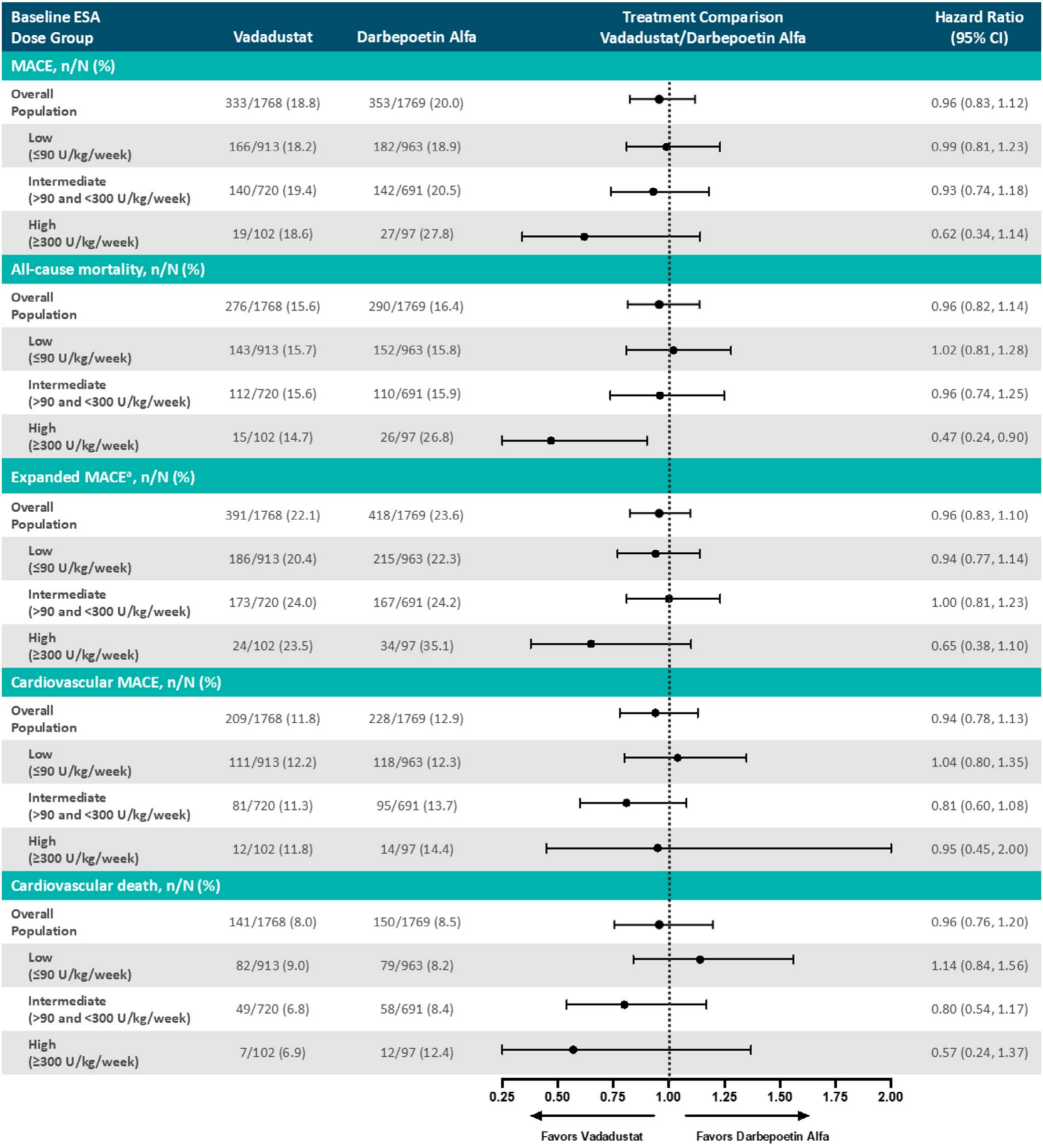
Prespecified MACE analysis by baseline ESA dose in patients with prevalent DD‐CKD (safety population). Any MACE plus hospitalizations for heart failure or thromboembolic events excluding vascular access thrombosis. DD‐CKD, dialysis‐dependent chronic kidney disease; ESA, erythropoiesis‐stimulating agent; MACE, major adverse cardiovascular events.

Among patients receiving low, intermediate, and high baseline ESA doses, a first MACE occurred in 18.2%, 19.4%, and 18.6% of vadadustat‐treated patients, and 18.9%, 20.5%, and 27.8% of darbepoetin‐treated patients, respectively (Figure 1, Table S1). Hazard ratios for MACE were 0.99, 95% CI, 0.81–1.23 (low [≤ 90 U/kg/week]); 0.93, 95% CI, 0.74–1.18 (intermediate [> 90 and < 300 U/kg/week]); and 0.62, 95% CI, 0.34–1.14 (high [≥ 300 U/kg/week]) (Figure 1). Treatment was not a significant factor for MACE (interaction *p* = 0.92); however, baseline ESA dose was a statistically significant risk factor for MACE (*p* = 0.04). Similar MACE results were observed for other endpoints, including all‐cause mortality, MACE plus hospitalizations for heart failure or thromboembolic events, cardiovascular MACE, and cardiovascular death (Figure 1, Table S1).

### 4.3 Treatment‐Emergent Serious Adverse Events of Special Interest

Treatment‐emergent serious adverse events of special interest were lower in vadadustat‐treated patients compared with darbepoetin‐treated patients across baseline ESA dose subgroups (Table 2). The difference in the proportion of patients with any treatment‐emergent serious adverse event of special interest between the vadadustat and darbepoetin groups was greatest in the high baseline ESA dose subgroup (48 patients [43.2%] in the vadadustat group and 57 patients [55.3%] in the darbepoetin group; relative risk, 0.78, 95% CI, 0.59–1.03) (Table 2).

**TABLE 2:** Treatment‐emergent serious adverse events of special interest by ESA baseline dose in patients with prevalent DD‐CKD (safety population).

Event, *n* (%)	≤ 90 U/kg/week			> 90 and < 300 U/kg/week			≥ 300 U/kg/week		
	Vadadustat (*n* = 949)	Darbepoetin alfa (*n* = 992)	RR (95% CI)	Vadadustat (*n* = 764)	Darbepoetin alfa (*n* = 738)	RR (95% CI)	Vadadustat (*n* = 111)	Darbepoetin alfa (*n* = 103)	RR (95% CI)
Any treatment‐emergent serious adverse events of special interest	337 (35.5)	396 (39.9)	0.89 (0.79, 1.00)	312 (40.8)	338 (45.8)	0.89 (0.79, 1.00)	48 (43.2)	57 (55.3)	0.78 (0.59, 1.03)
Hypertension	122 (12.9)	165 (16.6)	0.77 (0.62, 0.96)	144 (18.8)	159 (21.5)	0.88 (0.72, 1.07)	25 (22.5)	35 (34.0)	0.66 (0.43, 1.03)
Congestive heart failure	77 (8.1)	97 (9.8)	0.83 (0.62, 1.10)	85 (11.1)	92 (12.5)	0.89 (0.68, 1.18)	14 (12.6)	17 (16.5)	0.76 (0.40, 1.47)
Hyperkalemia	70 (7.4)	98 (9.9)	0.75 (0.56, 1.00)	75 (9.8)	84 (11.4)	0.86 (0.64, 1.16)	15 (13.5)	21 (20.4)	0.66 (0.36, 1.21)
Hypersensitivity^a^	70 (7.4)	85 (8.6)	0.86 (0.64, 1.17)	64 (8.4)	61 (8.3)	1.01 (0.72, 1.42)	9 (8.1)	7 (6.8)	1.19 (0.46, 3.09)
Hepatotoxicity	60 (6.3)	59 (5.9)	1.06 (0.75, 1.51)	53 (6.9)	64 (8.7)	0.80 (0.56, 1.13)	14 (12.6)	10 (9.7)	1.30 (0.60, 2.79)
Malignant or unspecified tumors	26 (2.7)	37 (3.7)	0.74 (0.45, 1.20)	17 (2.2)	22 (3.0)	0.75 (0.40, 1.39)	3 (2.7)	2 (1.9)	1.39 (0.24, 8.16)
Pulmonary hypertension	27 (2.8)	27 (2.7)	1.04 (0.62, 1.77)	20 (2.6)	25 (3.4)	0.77 (0.43, 1.38)	2 (1.8)	3 (2.9)	0.62 (0.11, 3.63)
Cardiac valve disorders	20 (2.1)	26 (2.6)	0.80 (0.45, 1.43)	22 (2.9)	26 (3.5)	0.82 (0.47, 1.43)	3 (2.7)	2 (1.9)	1.39 (0.24, 8.16)
Retinal disorders^b^	21 (2.2)	15 (1.5)	1.46 (0.76, 2.82)	11 (1.4)	16 (2.2)	0.66 (0.31, 1.42)	0 (0)	1 (1.0)	—
Adrenal disorder	1 (0.1)	1 (0.1)	1.05 (0.07, 16.69)	1 (0.1)	0 (0)	—	0 (0)	1 (1.0)	—

Abbreviations: DD‐CKD, dialysis‐dependent chronic kidney disease; ESA, erythropoiesis‐stimulating agent; RR, relative risk.

^a^ The preferred terms for hypersensitivity included allergic cough, allergic respiratory disease, allergic sinusitis, anaphylactic reaction, anaphylactic shock, antineutrophil cytoplasmic antibody‐positive vasculitis, angioedema, bronchospasm, catheter site eczema, catheter site rash, circulatory collapse, conjunctivitis allergic, contrast media allergy, contrast media reaction, corneal edema, cutaneous vasculitis, dermatitis, dermatitis allergic, dermatitis atopic, dermatitis bullous, dermatitis contact, dermatitis exfoliative, distributive shock, drug hypersensitivity, eczema, epidermolysis, eye allergy, eye edema, eye swelling, face edema, heparin‐induced thrombocytopenia, hypersensitivity, hypersensitivity pneumonitis, hypersensitivity vasculitis, iodine allergy, laryngeal edema, lip edema, lip swelling, periorbital edema, periorbital swelling, pharyngeal edema, procedural shock, pruritus allergic, rash, rash erythematous, rash maculo‐papular, rash pruritic, rash vesicular, rhinitis allergic, shock, skin reaction, skin necrosis, Stevens‐Johnson syndrome, swelling face, swelling of eyelid, swollen tongue, urticaria, urticaria cholinergic, vulval ulceration.

^b^ The preferred terms for retinal disorders included amaurosis fugax, cystoid macular edema, diabetic retinal edema, diabetic retinopathy, macular degeneration, macular edema, macular fibrosis, macular hole, retinal artery thrombosis, retinal detachment, retinal hemorrhage, retinal tear, retinal vascular disorder, retinal vein occlusion, retinopathy, retinopathy hypertensive, tractional retinal detachment, vitreous floaters, vitreous hemorrhage.

### 4.4 Changes in Hemoglobin Concentration

**FIGURE 2:**
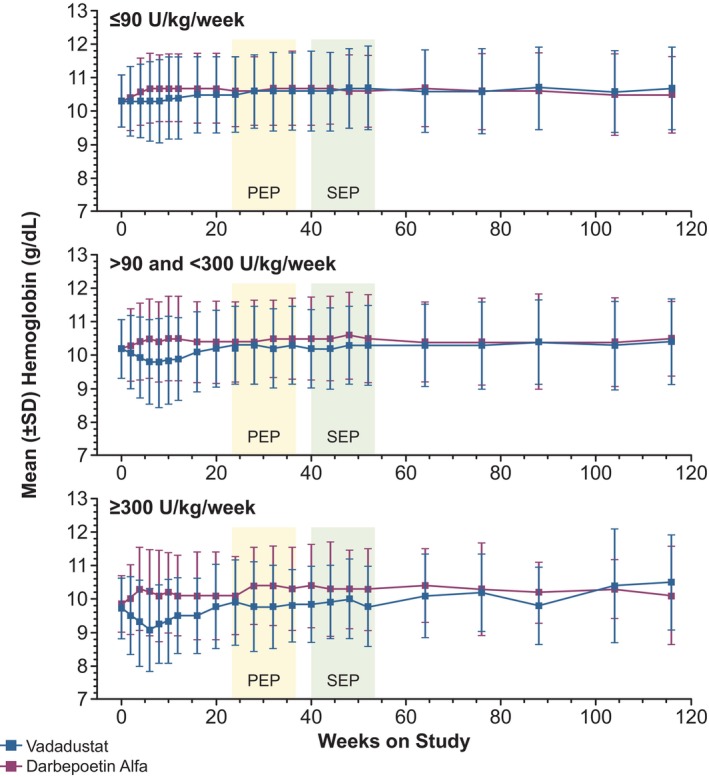
Mean hemoglobin over time by baseline ESA dose in patients with prevalent DD‐CKD. Geography‐specific target range for hemoglobin: 10–11 g/dL in the USA and 10–12 g/dL in other countries. DD‐CKD, dialysis‐dependent chronic kidney disease; ESA, erythropoiesis‐stimulating agent; PEP, primary evaluation period; SEP, secondary evaluation period.

Maintenance of hemoglobin with vadadustat was noninferior to darbepoetin across baseline ESA dose subgroups (Figure 2, Table S2). In vadadustat‐treated patients, a transient decline in mean hemoglobin was observed in the high baseline ESA dose subgroup during weeks 2–8, after which hemoglobin steadily increased by weeks 24–36. A less pronounced transient decline was observed in the intermediate baseline ESA dose subgroup, but not in the low baseline ESA dose subgroup (Figure 2).

The treatment difference (vadadustat–darbepoetin) for LS mean change in hemoglobin from baseline to the PEP was −0.10 g/dL (95% CI, −0.19 to −0.02), −0.20 g/dL (95% CI, −0.30 to −0.09), and −0.39 g/dL (95% CI, −0.67 to −0.11) for low, intermediate, and high baseline ESA dose subgroups, respectively (Table S2). Similar results were observed for the SEP (Table S2). In the analysis of covariance model with the interactions between ESA baseline dose and treatment groups, we found negative parameter estimations for both baseline ESA dose and the interaction terms in both the PEP (interaction term −0.00067, *p* = 0.03) and the SEP (interaction term −0.00121, *p* = 0.0002). This result suggests that the higher the baseline ESA dose, the less the change in hemoglobin from baseline.

The proportions of patients with mean hemoglobin within geography‐specific target range during the PEP and SEP were similar across treatment groups (Table 3). Response rates were higher in lower baseline ESA dose subgroups, irrespective of treatment, but were similar between treatment groups within each baseline ESA dose subgroup.

**TABLE 3:** Proportion of patients with average hemoglobin value within geography‐specific target range (responders) by baseline ESA dose during weeks 24–36 and 40–52 in patients with prevalent DD‐CKD (randomized population).

Subgroups by ESA dose at baseline	≤ 90 U/kg/week		> 90 and < 300 U/kg/week		≥ 300 U/kg/week	
	Vadadustat (*n* = 916)	Darbepoetin alfa (*n* = 968)	Vadadustat (*n* = 724)	Darbepoetin alfa (*n* = 693)	Vadadustat (*n* = 102)	Darbepoetin alfa (*n* = 98)
Weeks 24–36 (PEP)						
Responders based on the observed data, *n* (%; 95% CI)	481 (52.5) (49.2, 55.8)	544 (56.2) (53.0, 59.4)	341 (47.1) (43.4, 50.8)	354 (51.1) (47.3, 54.9)	39 (38.2) (28.8, 48.4)	40 (40.8) (31.0, 51.2)
Proportion difference (vadadustat–darbepoetin alfa) (95% CI)^a^	−0.027 (−0.072, 0.019)		−0.034 (−0.088, 0.021)		−0.027 (−0.175, 0.122)	
Weeks 40–52 (SEP)						
Responders based on the observed data, *n* (%; 95% CI)	439 (47.9) (44.7, 51.2)	503 (52.0) (48.8, 55.2)	301 (41.6) (38.0, 45.3)	358 (51.7) (47.9, 55.4)	31 (30.4) (21.7, 40.3)	33 (33.7) (24.4, 43.9)
Proportion difference (vadadustat–darbepoetin alfa) (95% CI)^a^	−0.031 (−0.078, 0.015)		−0.090 (−0.144, −0.036)		−0.039 (−0.186, 0.108)	

*Note:* Geography‐specific target range for hemoglobin: 10–11 g/dL in the USA and 10–12 g/dL in other countries.

Abbreviations: DD‐CKD, dialysis‐dependent chronic kidney disease; ESA, erythropoiesis‐stimulating agent; PEP, primary evaluation period; SEP, secondary evaluation period.

^a^ From Cochran‐Mantel‐Haenszel method by the 3 randomization stratification factors. When analyzing a subgroup that is itself a stratification variable, it is removed from the analysis model. Within any stratum, if there are no patients in any treatment group or no responders in both treatment groups, the unstratified Cochran‐Mantel‐Haenszel method is used instead for analysis.

### 4.5 Changes in Treatment Dose

#### 4.5.1 Daily Vadadustat Dose

**FIGURE 3:**
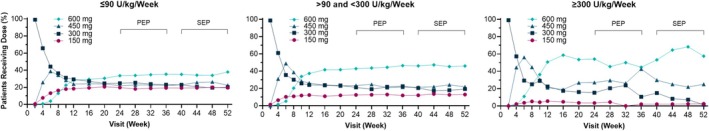
Daily vadadustat dose over the primary and secondary evaluation periods by baseline ESA dose (safety population). This analysis includes only those patients who were receiving vadadustat and not those whose doses were interrupted due to elevated hemoglobin levels, ESA rescue, or adverse events. ESA, erythropoiesis‐stimulating agent; PEP, primary evaluation period; SEP, secondary evaluation period.

All patients were initiated on vadadustat at 300 mg/day. By week 12, 52.3%, 63.6%, and 71.8% of patients were increased to 450 or 600 mg/day in the low, intermediate, and high baseline ESA dose subgroups, respectively. In the high baseline group, > 50% were receiving 600 mg/day (Figure 3). During both the PEP and SEP, these percentages increased further, especially in the high ESA dose subgroup (Figure 3).

#### 4.5.2 Weekly Vadadustat and Darbepoetin Doses

Mean (SD) vadadustat doses received were 2.42 (1.27), 2.62 (1.24), and 2.61 (1.33) g/week in the low, intermediate, and high baseline ESA subgroups, respectively, during the PEP, and 2.48 (1.24), 2.73 (1.21), and 2.83 (1.10) g/week, respectively, during the SEP. Mean (SD) darbepoetin doses received were 27.3 (23.9), 41.7 (34.3), and 49.6 (34.0) μg/week in the low, intermediate, and high ESA baseline subgroups, respectively, during the PEP, and 28.6 (26.4), 43.5 (36.2), and 47.0 (38.1) μg/week during the SEP.

### 4.6 Rescue Therapy

A higher proportion of patients in the darbepoetin treatment arm received ESA rescue in the low baseline ESA dose subgroup, whereas a higher proportion of patients in the vadadustat‐treated group received ESA rescue in the intermediate and high baseline ESA dose subgroups (Table S3). Among patients in intermediate and high baseline ESA dose subgroups, RBC transfusions were similar between treatment arms across all time points. In the low baseline ESA dose subgroup, RBC transfusion rescue was marginally higher for vadadustat‐treated patients than for darbepoetin‐treated patients at earlier time points (< 36 weeks) but similar between the treatment arms thereafter (Table S3).

### 4.7 Iron‐Related and Inflammation‐Related Parameters

Table 4 shows serum iron‐related and inflammation parameters. TIBC increased with vadadustat treatment during the PEP and SEP across all baseline ESA dose subgroups. Compared with these TIBC increases, relatively smaller changes occurred in serum iron and transferrin saturation across all ESA dose subgroups and between treatment groups from baseline to the PEP and SEP. Serum hepcidin decreased from baseline during the PEP and SEP in both treatment groups across all baseline ESA dose subgroups, with a trend for more reduction in vadadustat‐treated subgroups. The difference in mean serum hepcidin between vadadustat‐ and darbepoetin‐treated groups was greatest in the low baseline ESA dose subgroups (−31.9 ng/mL; 95% CI, −42.5 to −21.2). Serum ferritin was generally decreased from baseline during the PEP. Change from baseline in median serum ferritin during the PEP was most pronounced in the low baseline ESA dose subgroups (vadadustat: −73.3 ng/mL [lower quartile (Q1) to upper quartile (Q3), −246.8 to 110.0]; darbepoetin: −35.0 ng/mL [Q1 to Q3, −200.0 to 176.0]) with smaller changes among patients from the higher baseline ESA dose subgroups (Table S4). Median serum C‐reactive protein did not materially change in any baseline ESA dose subgroup (Table S4). Mean baseline C‐reactive protein was elevated in all subgroups, due to exceptionally elevated C‐reactive protein levels in a few patients in each subgroup (Table 4).

**TABLE 4:** Changes in iron‐related outcomes and C‐reactive protein by baseline ESA dose during weeks 24–36 and 40–52 in patients with prevalent DD‐CKD (randomized population).

Parameter (reference range)	Baseline		PEP (weeks 24–36)			SEP (weeks 40–52)		
	Vadadustat, mean (SD)	Darbepoetin alfa, mean (SD)	Vadadustat, ∆LS mean from baseline (SEM)	Darbepoetin alfa, ∆LS mean from baseline (SEM)	Difference (vadadustat–darbepoetin alfa), LS mean (95% CI)	Vadadustat, ∆LS mean from baseline (SEM)	Darbepoetin alfa, ∆LS mean from baseline (SEM)	Difference (vadadustat–darbepoetin alfa), LS mean (95% CI)
Baseline ESA dose subgroup: ≤ 90 U/kg/week								
TIBC, μg/dL (250–425 μg/dL^a^)	*n* = 915 211.9 (34.9)	*n* = 968 213.3 (35.4)	*n* = 803 29.7 (1.4)	*n* = 875 1.4 (1.3)	28.3 (25.5, 31.1)	*n* = 748 27.2 (1.5)	*n* = 825 0.3 (1.4)	26.8 (23.8, 29.8)
Hepcidin, ng/mL (male: 8.6–82.6 ng/mL; female: 6.2–51.5 ng/mL^a^)	*n* = 907 198.8 (138.9)	*n* = 952 194.1 (132.3)	*n* = 783 −61.1 (5.1)	*n* = 838 −29.2 (5.0)	−31.9 (−42.5, −21.2)	*n* = 648 −75.7 (5.4)	*n* = 703 −48.8 (5.3)	−26.9 (−37.9, −16.0)
Ferritin, ng/mL (10–380 ng/mL^a^)	*n* = 916 876 (569)	*n* = 968 855 (527)	*n* = 804 −114.0 (16.4)	*n* = 875 −57.1 (16.1)	−56.9 (−90.8, −23.1)	*n* = 748 −146.6 (17.3)	*n* = 827 −85.3 (17.0)	−61.3 (−96.7, −25.9)
Serum iron, μg/dL (male: 65–175; female: 50–170^a^)	*n* = 916 82.1 (28.3)	*n* = 968 80.6 (27.4)	*n* = 804 −2.4 (1.2)	*n* = 875 −7.3 (1.1)	4.9 (2.6, 7.3)	*n* = 748 −5.7 (1.3)	*n* = 826 −9.1 (1.3)	3.4 (0.7, 6.0)
Transferrin saturation, % (male: 20–50; female: 15–50^a^)	*n* = 915 39.2 (13.5)	*n* = 968 38.1 (13.1)	*n* = 803 −5.3 (0.5)	*n* = 875 −3.3 (0.5)	−2.0 (−3.1, −0.8)	*n* = 748 −6.6 (0.6)	*n* = 825 −4.0 (0.6)	−2.6 (−3.9, −1.4)
C‐reactive protein, mg/L (0.0–4.9 mg/L^a^)	*n* = 904 9.2 (17.9)	*n* = 947 9.4 (17.6)	*n* = 525 0.6 (1.0)	*n* = 552 1.7 (1.0)	−1.1 (−3.3, 1.0)	*n* = 638 1.3 (1.1)	*n* = 700 2.6 (1.1)	−1.3 (−3.6, 0.1)
Baseline ESA dose subgroup: > 90 and < 300 U/kg/week								
TIBC, μg/dL (250–425 μg/dL^a^)	*n* = 723 207.3 (36.4)	*n* = 693 210.1 (36.5)	*n* = 626 32.6 (2.0)	*n* = 615 1.7 (1.9)	31.0 (27.2, 34.7)	*n* = 562 29.6 (2.2)	*n* = 564 2.6 (2.1)	27.0 (22.9, 31.1)
Hepcidin, ng/mL (male: 8.6–82.6 ng/mL; female: 6.2–51.5 ng/mL^a^)	*n* = 715 191.9 (140.9)	*n* = 686 187.5 (139.6)	*n* = 612 −43.5 (6.3)	*n* = 585 −37.1 (6.3)	−6.4 (−18.6, 5.7)	*n* = 489 −57.3 (7.4)	*n* = 482 −32.8 (7.4)	−24.5 (−38.4, −10.6)
Ferritin, ng/mL (10–380 ng/mL^a^)	*n* = 723 824 (563)	*n* = 693 829 (560)	*n* = 628 −38.6 (25.8)	*n* = 618 −11.3 (25.8)	−27.2 (−77.0, 22.5)	*n* = 564 −54.7 (24.4)	*n* = 567 −7.9 (23.9)	−46.7 (−92.7, −0.7)
Serum iron, μg/dL (male: 65–175; female: 50–170^a^)	*n* = 723 77.3 (30.0)	*n* = 693 77.3 (28.0)	*n* = 627 2.7 (1.7)	*n* = 617 −4.4 (1.7)	7.1 (3.8, 10.3)	*n* = 563 0.3 (1.7)	*n* = 567 −2.4 (1.7)	2.7 (−0.6, 6.0)
Transferrin saturation, % (male: 20–50; female: 15–50^a^)	*n* = 723 37.2 (13.3)	*n* = 693 37.0 (12.9)	*n* = 626 −3.5 (0.7)	*n* = 615 −2.1 (0.7)	−1.3 (−2.7, 0.1)	*n* = 562 −3.8 (0.8)	*n* = 564 −1.2 (0.8)	−2.6 (−4.2, −1.1)
C‐reactive protein, mg/L (0.0–4.9 mg/L^a^)	*n* = 716 11.3 (22.9)	*n* = 680 9.5 (16.3)	*n* = 403 3.9 (1.3)	*n* = 395 3.5 (1.3)	0.4 (−2.1, 2.9)	*n* = 477 0.8 (1.6)	*n* = 475 3.0 (1.6)	−2.2 (−5.2, 0.8)
Baseline ESA dose subgroup: ≥ 300 U/kg/week								
TIBC, μg/dL (250–425 μg/dL^a^)	*n* = 101 209.1 (42.5)	*n* = 98 202.7 (41.4)	*n* = 78 36.4 (7.0)	*n* = 83 10.6 (7.2)	25.7 (16.6, 34.8)	*n* = 75 40.1 (7.8)	*n* = 80 10.2 (7.9)	29.9 (19.8, 40.0)
Hepcidin, ng/mL (male: 8.6–82.6 ng/mL; female: 6.2–51.5 ng/mL^a^)	*n* = 102 173.8 (144.0)	*n* = 96 178.9 (146.1)	*n* = 74 −82.2 (27.8)	*n* = 77 −74.3 (28.1)	−8.0 (−44.0, 28.1)	*n* = 63 −43.4 (28.5)	*n* = 66 −63.3 (28.0)	20.0 (−14.3, 54.2)
Ferritin, ng/mL (10–380 ng/mL^a^)	*n* = 102 798 (520)	*n* = 98 832 (496)	*n* = 79 −40.1 (82.1)	*n* = 83 −51.3 (83.5)	11.2 (−92.9, 115.3)	*n* = 76 27.5 (94.5)	*n* = 80 −68.0 (96.3)	95.5 (−25.7, 216.7)
Serum iron, μg/dL (male: 65–175; female: 50–170^a^)	*n* = 102 75.9 (33.3)	*n* = 98 75.0 (33.7)	*n* = 79 5.9 (7.3)	*n* = 83 1.6 (7.4)	4.3 (−5.1, 13.6)	*n* = 76 8.2 (6.5)	*n* = 80 2.7 (6.6)	5.5 (−2.9, 13.9)
Transferrin saturation, % (male: 20–50; female: 15–50^a^)	*n* = 101 35.9 (13.7)	*n* = 98 37.5 (15.4)	*n* = 78 −1.6 (3.2)	*n* = 83 0.9 (3.3)	−2.5 (−6.7, 1.7)	*n* = 75 −1.5 (2.9)	*n* = 80 0.7 (2.9)	−2.2 (−6.0, 1.5)
C‐reactive protein, mg/L (0.0–4.9 mg/L^a^)	*n* = 102 11.6 (16.8)	*n* = 96 13.4 (21.5)	*n* = 49 −0.6 (4.3)	*n* = 53 2.8 (4.3)	−3.4 (−9.8, 3.0)	*n* = 61 1.8 (6.0)	*n* = 67 −0.8 (6.0)	2.6 (−5.4, 10.6)

Abbreviations: ∆, change (from baseline); DD‐CKD, dialysis‐dependent chronic kidney disease; ESA, erythropoiesis‐stimulating agent; LS, least‐squares; PEP, primary evaluation period; SEP; secondary evaluation period; TIBC, total iron‐binding capacity.

^a^ Reference ranges [32, 33].

### 4.8 Serum EPO, Reticulocytes, and RBC Indices

Table 5 shows mean serum EPO concentrations measured before administration of vadadustat or darbepoetin at baseline, 28 weeks, and 52 weeks (includes darbepoetin subgroups, whose EPO receptors are stimulated by both endogenous EPO and darbepoetin). Baseline EPO levels were in the range of healthy individuals for low ESA dose subgroups, very slightly increased above normal for intermediate subgroups, and slightly further elevated in high subgroups. EPO levels remained in these respective ranges during the trial (Table 5). Reticulocytes/μL in all vadadustat‐ and darbepoetin‐treated subgroups were maintained in the normal range for healthy individuals but less than expected for the same degree of anemia in healthy individuals with intact erythropoietic responses (Table 5). Compared with their respective low and intermediate subgroups, both high ESA dose subgroups had slightly increased baseline reticulocytes, but similar reticulocytes at 28 and 52 weeks (Table 5). Reticulocytes in each vadadustat‐treated subgroup were very slightly increased, as previously noted, compared with their respective darbepoetin‐treated subgroups (Table 5). Steady‐state RBCs/μL were essentially stable in all subgroups throughout the trial (Table 5). Baseline mean corpuscular volume and mean corpuscular hemoglobin of patients' RBCs were at the upper end of the normal ranges in all subgroups, where they remained throughout the study (Table 6).

**TABLE 5:** Changes in EPO, reticulocytes, and RBCs from baseline by ESA baseline dose in patients with prevalent DD‐CKD (randomized population).

Parameter (reference range)	≤ 90 U/kg/week		> 90 and < 300 U/kg/week		≥ 300 U/kg/week	
	Vadadustat (*n* = 916)	Darbepoetin alfa (*n* = 968)	Vadadustat (*n* = 724)	Darbepoetin alfa (*n* = 693)	Vadadustat (*n* = 102)	Darbepoetin alfa (*n* = 98)
EPO, mIU/mL, mean (SD) (4–26 mIU/mL^a^)						
Baseline	*n* = 906 19.7 (35.2)	*n* = 950 20.8 (64.5)	*n* = 713 28.9 (86.4)	*n* = 685 31.8 (89.2)	*n* = 102 66.4 (182.8)	*n* = 97 43.7 (93.2)
Week 28 (PEP)	*n* = 780 19.5 (46.4)	*n* = 844 23.3 (143.3)	*n* = 607 26.1 (56.3)	*n* = 581 32.6 (78.6)	*n* = 74 30.5 (66.3)	*n* = 79 52.1 (142.9)
Change from baseline at week 28	*n* = 773 0.8 (54.6)	*n* = 830 5.6 (147.3)	*n* = 602 −3.1 (104.9)	*n* = 573 −0.3 (117.1)	*n* = 74 −22.4 (126.6)	*n* = 78 5.8 (177.0)
Treatment difference (vadadustat–darbepoetin alfa), LS mean (95% CI); *p* value	−4.0 (−14.7, 6.6); *p =* 0.460		−6.0 (−13.8, 1.7); *p =* 0.128		−23.7 (−58.5, 11.1); *p =* 0.182	
Week 52 (SEP)	*n* = 628 23.7 (47.8)	*n* = 692 21.2 (67.3)	*n* = 461 39.8 (133.1)	*n* = 471 27.8 (62.1)	*n* = 65 63.6 (190.6)	*n* = 65 28.1 (39.8)
Change from baseline at week 52	*n* = 623 4.9 (56.4)	*n* = 681 3.1 (69.7)	*n* = 460 8.9 (156.9)	*n* = 466 −3.1 (110.3)	*n* = 65 9.4 (196.5)	*n* = 64 −24.5 (111.3)
Treatment difference (vadadustat–darbepoetin alfa), LS mean (95% CI); *p* value	2.5 (−3.9, 8.9); *p =* 0.443		11.4 (−1.9, 24.7); *p =* 0.092		34.3 (−12.6, 81.1); *p =* 0.152	
Reticulocytes, ×10^3^/μL, mean (SD) (25–100 × 10^3^/μL^a^)						
Baseline	*n* = 870 54.3 (28.1)	*n* = 911 54.2 (25.9)	*n* = 687 54.3 (29.3)	*n* = 669 54.9 (30.0)	*n* = 98 72.6 (40.6)	*n* = 98 64.4 (39.3)
Week 28 (PEP)	*n* = 768 55.5 (24.5)	*n* = 823 50.3 (26.8)	*n* = 599 55.4 (28.0)	*n* = 579 51.7 (29.8)	*n* = 76 60.9 (34.0)	*n* = 77 56.5 (36.6)
Change from baseline at week 28	*n* = 733 0.6 (29.0)	*n* = 778 −4.7 (31.0)	*n* = 572 1.6 (33.6)	*n* = 560 −2.3 (32.8)	*n* = 72 −11.8 (50.2)	*n* = 77 −9.8 (44.7)
Treatment difference (vadadustat–darbepoetin alfa), LS mean (95% CI); *p* value	5.2 (2.8, 7.6); *p* < 0.0001		3.9 (0.8, 7.1); *p =* 0.015		3.1 (−7.9, 14.1); *p =* 0.586	
Week 52 (SEP)	*n* = 621 53.9 (25.7)	*n* = 692 46.8 (24.2)	*n* = 459 55.8 (31.3)	*n* = 473 46.8 (28.9)	*n* = 67 52.7 (27.8)	*n* = 66 49.4 (31.1)
Change from baseline at week 52	*n* = 590 −1.9 (30.8)	*n* = 654 −7.5 (28.4)	*n* = 440 1.0 (37.5)	*n* = 462 −8.5 (32.8)	*n* = 63 −18.4 (42.7)	*n* = 66 −16.1 (42.0)
Treatment difference (vadadustat–darbepoetin alfa), LS mean (95% CI); *p* value	6.6 (4.1, 9.2); *p* < 0.0001		9.0 (5.3, 12.7); *p* < 0.0001		1.6 (−8.0, 11.2); *p =* 0.744	
RBCs, ×10^6^/μL, mean (SD) (4.2–5.9 × 10^6^/μL^a^)						
Baseline	*n* = 916 3.3 (0.4)	*n* = 968 3.3 (0.4)	*n* = 724 3.3 (0.4)	*n* = 693 3.3 (0.4)	*n* = 102 3.2 (0.4)	*n* = 98 3.2 (0.4)
Week 28 (PEP)	*n* = 775 3.4 (0.4)	*n* = 839 3.4 (0.4)	*n* = 604 3.3 (0.5)	*n* = 585 3.4 (0.5)	*n* = 77 3.1 (0.4)	*n* = 78 3.3 (0.4)
Change from baseline at week 28	*n* = 775 0.1 (0.4)	*n* = 839 0.1 (0.4)	*n* = 604 0.0 (0.5)	*n* = 585 0.1 (0.5)	*n* = 77 −0.1 (0.5)	*n* = 78 0.1 (0.5)
Treatment difference (vadadustat–darbepoetin alfa), LS mean (95% CI); *p* value	−0.1 (−0.1, 0.0); *p =* 0.0014		−0.1 (−0.2, −0.1); *p* < 0.0001		−0.2 (−0.4, −0.1); *p =* 0.0001	
Week 52 (SEP)	*n* = 646 3.4 (0.5)	*n* = 718 3.4 (0.4)	*n* = 494 3.3 (0.5)	*n* = 498 3.4 (0.5)	*n* = 71 3.1 (0.5)	*n* = 71 3.3 (0.4)
Change from baseline at week 52	*n* = 646 0.1 (0.5)	*n* = 718 0.1 (0.4)	*n* = 494 0.0 (0.5)	*n* = 498 0.1 (0.5)	*n* = 71 −0.1 (0.5)	*n* = 71 0.1 (0.5)
Treatment difference (vadadustat–darbepoetin alfa), LS mean (95% CI); *p* value	0.0 (−0.1, 0.0); *p =* 0.385		−0.1 (−0.2, −0.1); *p* < 0.0001		−0.2 (−0.3, 0.0); *p =* 0.017	

Abbreviations: DD‐CKD, dialysis‐dependent chronic kidney disease; EPO, erythropoietin; ESA, erythropoiesis‐stimulating agent; LS, least‐squares; PEP, primary evaluation period; RBCs, red blood cells; SEP; secondary evaluation period.

^a^ Reference ranges [33].

**TABLE 6:** Changes in RBC indices from baseline by ESA baseline dose in patients with prevalent DD‐CKD (randomized population).

Parameter (reference range)	≤ 90 U/kg/week		> 90 and < 300 U/kg/week		≥ 300 U/kg/week	
	Vadadustat (*n* = 916)	Darbepoetin alfa (*n* = 968)	Vadadustat (*n* = 724)	Darbepoetin alfa (*n* = 693)	Vadadustat (*n* = 102)	Darbepoetin alfa (*n* = 98)
Mean corpuscular volume, fL, mean (SD) (79–98 fL^a^)						
Baseline	*n* = 916 95.0 (6.4)	*n* = 968 94.9 (5.7)	*n* = 724 94.8 (7.4)	*n* = 693 94.6 (7.1)	*n* = 102 94.8 (7.2)	*n* = 98 95.3 (6.6)
Week 28 (PEP)	*n* = 772 96.2 (6.6)	*n* = 837 95.6 (6.2)	*n* = 602 96.1 (7.5)	*n* = 585 95.1 (7.3)	*n* = 77 96.2 (7.3)	*n* = 78 96.4 (5.9)
Change from baseline at week 28	*n* = 772 1.2 (4.3)	*n* = 837 0.8 (4.1)	*n* = 602 1.2 (5.2)	*n* = 585 0.6 (4.4)	*n* = 77 1.5 (4.4)	*n* = 78 0.9 (4.1)
Treatment difference (vadadustat–darbepoetin alfa), LS mean (95% CI); *p* value	0.5 (0.1, 0.9); *p =* 0.017		0.6 (0.1, 1.2); *p =* 0.02		0.5 (−0.8, 1.8); *p =* 0.435	
Week 52 (SEP)	*n* = 643 96.0 (6.7)	*n* = 717 95.6 (6.3)	*n* = 490 96.3 (7.5)	*n* = 498 95.4 (7.5)	*n* = 71 95.9 (8.6)	*n* = 71 97.6 (6.8)
Change from baseline at week 52	*n* = 643 1.2 (4.3)	*n* = 717 0.9 (4.1)	*n* = 490 1.5 (5.3)	*n* = 498 0.7 (5.0)	*n* = 71 1.2 (5.5)	*n* = 71 1.9 (5.1)
Treatment difference (vadadustat–darbepoetin alfa), LS mean (95% CI); *p* value	0.3 (−0.1, 0.8); *p =* 0.130		0.8 (0.2, 1.4); *p =* 0.008		−0.9 (−2.6, 0.8); *p =* 0.282	
Mean corpuscular hemoglobin, pg/cell, mean (SD) (26–34 pg/cell^a^)						
Baseline	*n* = 916 31.3 (2.2)	*n* = 968 31.3 (2.1)	*n* = 724 30.9 (2.6)	*n* = 693 31.0 (2.6)	*n* = 102 30.8 (2.7)	*n* = 98 30.9 (2.4)
Week 28 (PEP)	*n* = 775 31.5 (2.3)	*n* = 839 31.3 (2.2)	*n* = 604 31.5 (2.7)	*n* = 585 31.0 (2.7)	*n* = 77 31.7 (2.6)	*n* = 78 31.5 (2.2)
Change from baseline at week 28	*n* = 775 0.3 (1.4)	*n* = 839 0.1 (1.4)	*n* = 604 0.5 (1.7)	*n* = 585 0.0 (1.5)	*n* = 77 1.0 (1.6)	*n* = 78 0.4 (1.3)
Treatment difference (vadadustat–darbepoetin alfa), LS mean (95% CI); *p* value	0.2 (0.1, 0.4); *p =* 0.001		0.5 (0.3, 0.7); *p* < 0.0001		0.4 (0.0, 0.9); *p =* 0.046	
Week 52 (SEP)	*n* = 646 31.5 (2.4)	*n* = 718 31.3 (2.3)	*n* = 494 31.4 (2.8)	*n* = 498 31.0 (2.8)	*n* = 71 31.6 (3.2)	*n* = 71 31.6 (2.3)
Change from baseline at week 52	*n* = 646 0.2 (1.6)	*n* = 718 0.0 (1.4)	*n* = 494 0.5 (1.8)	*n* = 498 −0.1 (1.8)	*n* = 71 1.0 (2.0)	*n* = 71 0.5 (1.5)
Treatment difference (vadadustat–darbepoetin alfa), LS mean (95% CI); *p* value	0.2 (0.1, 0.4); *p =* 0.0104		0.6 (0.3, 0.8); *p* < 0.0001		0.3 (−0.2, 0.9); *p =* 0.237	
RBC distribution width, %, mean (SD) (9.0%–14.5%^a^)						
Baseline	*n* = 916 15.4 (1.6)	*n* = 968 15.4 (1.6)	*n* = 724 16.2 (2.0)	*n* = 693 16.2 (1.9)	*n* = 102 17.2 (2.1)	*n* = 98 17.0 (2.1)
Week 28 (PEP)	*n* = 772 15.2 (1.6)	*n* = 837 16.0 (1.8)	*n* = 602 15.5 (1.8)	*n* = 585 16.6 (2.1)	*n* = 77 16.0 (2.3)	*n* = 78 16.7 (2.3)
Change from baseline at week 28	*n* = 772 −0.3 (1.8)	*n* = 837 0.6 (1.8)	*n* = 602 −0.6 (2.3)	*n* = 585 0.5 (2.1)	*n* = 77 −1.2 (2.2)	*n* = 78 −0.3 (2.1)
Treatment difference (vadadustat–darbepoetin alfa), LS mean (95% CI); *p* value	−0.9 (−1.0, −0.7); *p* < 0.0001		−1.0 (−1.2, −0.8); *p* < 0.0001		−0.8 (−1.4, −0.2); *p =* 0.008	
Week 52 (SEP)	*n* = 643 15.4 (1.7)	*n* = 717 16.2 (1.8)	*n* = 490 15.9 (2.0)	*n* = 498 16.7 (2.0)	*n* = 71 16.1 (2.2)	*n* = 71 16.6 (2.3)
Change from baseline at week 52	*n* = 643 0.0 (1.8)	*n* = 717 0.8 (1.9)	*n* = 490 −0.3 (2.5)	*n* = 498 0.6 (2.1)	*n* = 71 −1.2 (2.0)	*n* = 71 −0.2 (2.6)
Treatment difference (vadadustat–darbepoetin alfa), LS mean (95% CI); *p* value	−0.8 (−1.0, −0.6); *p* < 0.0001		−0.9 (−1.1, −0.6); *p* < 0.0001		−0.8 (−1.4, −0.1); *p =* 0.022	

^a^ Reference ranges [33].

## 5 Discussion

When comparing safety and efficacy between vadadustat and darbepoetin by baseline ESA dose among patients with CKD on maintenance dialysis, we found no meaningful between‐group differences. No difference was found in the primary cardiovascular safety endpoint, and similar outcomes were noted for other safety endpoints, including all‐cause mortality, MACE plus hospitalizations for heart failure or thromboembolic events, and cardiovascular death. The vadadustat‐treated group had fewer treatment‐emergent serious adverse events of special interest across baseline ESA dose subgroups.

Vadadustat was noninferior (threshold of −0.75 g/dL) to darbepoetin for hemoglobin maintenance across the 3 baseline ESA dose subgroups. However, vadadustat‐treated patients requiring high and intermediate doses of ESAs at baseline had transient declines in mean hemoglobin concentration during weeks 2–8, followed by steady increases until week 20. The vadadustat low baseline ESA dose subgroup had no transient hemoglobin decrease. These results are consistent with initiating all vadadustat‐treated patients at 300 mg/day and limiting their maximum dose to 600 mg/day, while initial doses for darbepoetin‐treated patients were based on ESA dose at enrollment with no maximum dose. In each subgroup, vadadustat‐treated patients were frequently titrated up from the initiating 300 mg/day dose, suggesting that patients with higher ESA baseline doses may require higher initial and maintenance doses of vadadustat. Further clinical trials will be required to determine whether the use of higher vadadustat doses can avoid early transient declines in hemoglobin and sustain hemoglobin responses in patients like those in the high baseline ESA subgroup, without increasing the relatively low levels of MACE found here. Darbepoetin doses were not limited and, as expected for subgroups defined by baseline ESA dose, darbepoetin doses were proportionately greater as baseline ESA subgroups increased from low to high. More frequent ESA rescue in the darbepoetin‐treated low baseline ESA subgroup was likely due to darbepoetin dose adjustments not being limited and dose doubling being considered as rescue. More frequent ESA rescue in the vadadustat‐treated intermediate and high baseline ESA subgroups was likely due to the vadadustat maximum dose being limited to 600 mg/day.

The few differences in patient characteristics between the high baseline ESA dose subgroup and the low and intermediate baseline ESA dose subgroups—higher proportions of Black patients, lower hemoglobin and body mass index—have been associated with higher ESA requirements [34, 35, 36, 37].

Vadadustat increases iron availability compared with darbepoetin in DD‐CKD–related anemia, as increased serum transferrin (measured as TIBC) is accompanied by decreased transferrin saturation, serum hepcidin, and serum ferritin. In each ESA dose subgroup, vadadustat significantly increased TIBC from baseline during the PEP and SEP, an effect consistent with HIF‐mediated *transferrin* transcription [38] that was not observed in the respective darbepoetin‐treated subgroups. Serum hepcidin was reduced in all subgroups from baseline to the PEP, with more pronounced reductions in vadadustat‐treated patients, especially the low baseline ESA dose subgroup. Slightly to moderately elevated serum C‐reactive protein remaining essentially unchanged in all subgroups suggests that hepcidin decreases were less related to decreased inflammation than increased erythroferrone by vadadustat and darbepoetin enhancement of erythropoietic activity as well as vadadustat‐mediated *transferrin* transcription.

In vadadustat‐treated patients, mean endogenous EPO increased slightly with increasing ESA baseline doses but remained in the normal to slightly elevated range. These EPO concentrations resulted in reticulocytes/μL in the normal range, while steady‐state RBCs/μL remained in the targeted moderately anemic range. Endogenous EPO is slightly increased by vadadustat and other HIF‐PHIs, without the large transient increases in EPO seen with ESAs [23]. Because EPO prevents apoptotic death of erythroid progenitors [39], sustained normal to slightly elevated EPO levels can maintain regulated RBC production [40], as observed here in vadadustat‐treated patients.

Maintaining reticulocytes/μL in the normal range and total RBCs/μL in the moderately anemic range in each vadadustat and darbepoetin subgroup is consistent with reduced RBC lifespans that negatively correlate with ESA dose in hemodialyzed patients with CKD [41]. Relatively increased reticulocytes in the high ESA subgroup at baseline are most likely due to large ESA doses increasing reticulocytes but not RBC numbers because after the reticulocytes become RBCs their lifespan is the shortest of the 3 subgroups. Modeling of DD‐CKD patients' anemia with reduced RBC lifespans predicts increased endogenous EPO within the normal and slightly above‐normal range can achieve hemoglobin concentrations between 10 and 12 g/dL [42]. As baseline ESA dose increased, corresponding vadadustat dose increased, resulting in slightly increased endogenous EPO production while maintaining the target hemoglobin concentration. These results and vadadustat pharmacology [23] suggest that further increases in vadadustat doses will likely improve the responses of a range of patients, including those with high baseline ESA requirements, while only slightly elevating endogenous EPO.

Slightly increased mean corpuscular volume and mean corpuscular hemoglobin at 28 and 52 weeks contributed to the maintenance of hemoglobin in all subgroups and are consistent with increased iron availability due to reduced hepcidin. Slightly greater increases in vadadustat‐treated subgroups than in the respective darbepoetin‐treated subgroups, especially in the low and intermediate subgroups, suggest that mean corpuscular volume and mean corpuscular hemoglobin of the high baseline ESA dose subgroup might increase further if vadadustat doses were increased above 600 mg/day.

Limitations of our analyses include the restricted range of vadadustat doses and variable iron supplementation by individual investigator practices. Future studies including higher vadadustat doses and more regulated iron supplementation will provide information for vadadustat dose optimization. The present analyses, however, show that vadadustat provides regulated, limited inductions of endogenous EPO, improves iron availability, and represents a safe, effective alternative to the current treatment of patients with anemia and DD‐CKD, including patients requiring high ESA doses.

## Funding

This study was funded by Akebia Therapeutics Inc.

## Ethics Statement

Before initiating a study, each investigator received approval from their respective institutional review board and/or independent ethics committee. Study investigators obtained additional study approvals as required by national and local ethics regulations. The present manuscript is a secondary analysis of original research previously published in *The New England Journal of Medicine* [19] For a list of the study investigators who participated in the original phase 3 trials and therefore received institutional review board/independent ethics committee approval and written patient consent forms, please see the original research article [19].

## Consent

Study investigators obtained written informed patient consent as required by national and local ethics regulations. An independent ethics committee approved the informed consent forms.

## Conflicts of Interest

A.J. reported personal fees from Akebia Therapeutics, as a consultant and member of steering committees for phase 3 trials of vadadustat. S.K.B., W.L., and T.M. are employees of Akebia Therapeutics. M.J.S. reports consultancy on trial steering committees funded by Akebia Therapeutics, consultancy for Boehringer Ingelheim (attended an advisory board), and research funding from the National Institutes of Health. M.J.S.'s spouse reports employment with and ownership interest in Eli Lilly. W.C.W. holds the Gordon A. Cain Chair in Nephrology at Baylor College of Medicine and has served as a consultant for and received honoraria from Anthos, Akebia Therapeutics, Ardelyx, AstraZeneca, Bayer, Boehringer Ingelheim, GlaxoSmithKline, Merck Sharp & Dohme/Merck, Natera, Novartis, Pharmacosmos, Unicycive, Vera, and Zydus. R.A. has received consultancy fees from Vifor, Boehringer Ingelheim, Eli Lilly, Akebia Therapeutics, Reata, Diamedica, Bayer, Chinook, and Vertex. G.M.C. has served on the board of directors of Satellite Healthcare, a nonprofit dialysis provider. He has served as chair or co‐chair of trial steering committees with Akebia Therapeutics, AstraZeneca, CSL Behring, Sanifit, and Vertex. He has served as an advisor to Applaud, Ardelyx, Calico, CloudCath, Durect, Eliaz Therapeutics, Miromatrix, Outset, Renibus, and Unicycive. He has served on data safety monitoring boards with Bayer, Mineralys, and ReCor. He has received research grants from NIAID, NIDDK, and NHLBI. K.U.E. reports personal fees from Akebia Therapeutics as a member of the steering committee for phase 3 trials of vadadustat, lecture fees from Bayer and Boehringer Ingelheim, consulting fees from Novartis, and grant support from Evotec. He has served as a consultant for Akebia Therapeutics and has a grant/contract with Amgen, Bayer Healthcare, and Vifor Pharma. M.J.K. reports personal fees from Akebia Therapeutics as a consultant and member of steering and publication committees for phase 3 trials of vadadustat; from Alexion Pharmaceuticals as a consultant; and from GlaxoSmithKline as a consultant and member of its educators' network. He also is a contributor to BMJ Best Practice.

## Supporting information


**Table S1:** Detailed analysis of prespecified MACE by baseline ESA dose in patients with prevalent DD‐CKD (safety population).
**Table S2:** Change in hemoglobin from baseline for vadadustat treatment during primary and secondary evaluation periods by baseline ESA dose in patients with prevalent DD‐CKD (randomized population).
**Table S3:** Analysis of rescue therapy use by baseline ESA dose in patients with prevalent DD‐CKD.
**Table S4:** Changes in median values of ferritin and C‐reactive protein by baseline ESA dose during weeks 24–36 and 40–52 in patients with prevalent DD‐CKD (randomized population).

## Data Availability

Proposals for access to original data should be sent to medicalinfo@akebia.com. Deidentified patient‐level data will be available 12 months after USA and Europe approval to qualified researchers with an appropriate research proposal. The research proposal is subject to review by an institutional review board with final approval by Akebia Therapeutics Inc.
