# Supplementary material for: Safety and Efficacy of Vadadustat Versus Darbepoetin Alfa for Chronic Kidney Disease–Related Anemia in Patients Receiving Dialysis by Baseline Erythropoiesis‐Stimulating Agent Dose
Source: Hemodial Int. 2025 Dec 11;30(1):80–100. doi: 10.1111/hdi.70034 (PMC12817161; doi:10.1111/hdi.70034)
Supplement: Supplementary file 1 — Table S1: Detailed analysis of prespecified MACE by baseline ESA dose in patients with prevalent DD‐CKD (safety population). Table S2: Change in hemoglobin from baseline for vadadustat treatment during primary and secondary evaluation periods by baseline ESA dose in patients with prevalent DD‐CKD (randomized population). Table S3: Analysis of rescue therapy use by baseline ESA dose in patients with prevalent DD‐CKD. Table S4: Changes in median values of ferritin and C‐reactive protein by baseline ESA dose during weeks 24–36 and 40–52 in patients with prevalent DD‐CKD (randomized population). [file HDI-30-80-s001.docx]

**Supplementary Material for:**

Safety and Efficacy of Vadadustat Versus Darbepoetin Alfa for Chronic Kidney Disease-Related Anemia in Patients Receiving Dialysis by Baseline Erythropoiesis Stimulating Agent Dose

Jardine A, et al.

| **Supplementary Material** | **Title** | **Page** |
| --- | --- | --- |
| Table S1 | Detailed analysis of prespecified MACE by baseline ESA dose in patients with prevalent DD-CKD (safety population) | 2 |
| Table S2 | Change in hemoglobin from baseline for vadadustat treatment during primary and secondary evaluation periods by baseline ESA dose in patients with prevalent DD-CKD (randomized population) | 4 |
| Table S3 | Analysis of rescue therapy use by baseline ESA dose in patients with prevalent DD-CKD | 5 |
| Table S4 | Changes in median values of ferritin and C-reactive protein by baseline ESA dose during weeks 24-36 and 40-52 in patients with prevalent DD-CKD (randomized population) | 6 |
| References | | 7 |

**Table S1.** Detailed analysis of prespecified MACE by baseline ESA dose in patients with prevalent DD-CKD (safety population)

|  | **≤90 U/kg/wk** | | **>90 and <300 U/kg/wk** | | **≥300 U/kg/wk** | |
| --- | --- | --- | --- | --- | --- | --- |
|  | **Vadadustat  (n = 913)** | **Darbepoetin Alfa (n = 963)** | **Vadadustat (n = 720)** | **Darbepoetin Alfa (n = 691)** | **Vadadustat (n = 102)** | **Darbepoetin Alfa (n = 97)** |
| **Any MACE, n (%)** | 166 (18.2) | 182 (18.9) | 140 (19.4) | 142 (20.5) | 19 (18.6) | 27 (27.8) |
| All-cause mortality | 143 (15.7) | 152 (15.8) | 112 (15.6) | 110 (15.9) | 15 (14.7) | 26 (26.8) |
| Nonfatal MI | 35 (3.8) | 48 (5.0) | 34 (4.7) | 35 (5.1) | 6 (5.9) | 2 (2.1) |
| Nonfatal stroke | 13 (1.4) | 20 (2.1) | 12 (1.7) | 18 (2.6) | 2 (2.0) | 2 (2.1) |
| **All-cause mortality, n (%)** | 143 (15.7) | 152 (15.8) | 112 (15.6) | 110 (15.9) | 15 (14.7) | 26 (26.8) |
| CV death | 82 (9.0) | 79 (8.2) | 49 (6.8) | 58 (8.4) | 7 (6.9) | 12 (12.4) |
| Non-CV death | 49 (5.4) | 56 (5.8) | 53 (7.4) | 40 (5.8) | 3 (2.9) | 11 (11.3) |
| Unknown death | 12 (1.3) | 17 (1.8) | 10 (1.4) | 12 (1.7) | 5 (4.9) | 3 (3.1) |
| **Expanded MACE, n (%)** |  |  |  |  |  |  |
| Any MACE plus TE | 221 (24.2) | 245 (25.4) | 196 (27.2) | 175 (25.3) | 26 (25.5) | 33 (34.0) |
| Any MACE plus TE excluding VAT | 170 (18.6) | 192 (19.9) | 153 (21.3) | 149 (21.6) | 19 (18.6) | 27 (27.8) |
| Any MACE plus hospitalizations for HF | 182 (19.9) | 206 (21.4) | 161 (22.4) | 163 (23.6) | 24 (23.5) | 34 (35.1) |
| Any MACE plus hospitalizations for HF or TE | 233 (25.5) | 266 (27.6) | 214 (29.7) | 193 (27.9) | 29 (28.4) | 39 (40.2) |
| Any MACE plus hospitalizations for HF or TE excluding VAT | 186 (20.4) | 215 (22.3) | 173 (24.0) | 167 (24.2) | 24 (23.5) | 34 (35.1) |
| **Any CV MACE, n (%)** | 111 (12.2) | 118 (12.3) | 81 (11.3) | 95 (13.7) | 12 (11.8) | 14 (14.4) |
| CV death | 82 (9.0) | 79 (8.2) | 49 (6.8) | 58 (8.4) | 7 (6.9) | 12 (12.4) |
| Nonfatal MI | 35 (3.8) | 48 (5.0) | 34 (4.7) | 35 (5.1) | 6 (5.9) | 2 (2.1) |
| Nonfatal stroke | 13 (1.4) | 20 (2.1) | 12 (1.7) | 18 (2.6) | 2 (2.0) | 2 (2.1) |
| **CV death, n (%)** | 82 (9.0) | 79 (8.2) | 49 (6.8) | 58 (8.4) | 7 (6.9) | 12 (12.4) |
| **Any hospitalization for HF, n (%)** | 34 (3.7) | 40 (4.2) | 33 (4.6) | 33 (4.8) | 6 (5.9) | 9 (9.3) |
| **Any TE, n (%)** | 79 (8.7) | 83 (8.6) | 71 (9.9) | 45 (6.5) | 11 (10.8) | 7 (7.2) |
| Vascular access thrombosis | 70 (7.7) | 71 (7.4) | 57 (7.9) | 33 (4.8) | 11 (10.8) | 7 (7.2) |
| Arterial thrombosis | 3 (0.3) | 3 (0.3) | 4 (0.6) | 1 (0.1) | 0 (0.0) | 0 (0.0) |
| Deep vein thrombosis | 5 (0.5) | 9 (0.9) | 10 (1.4) | 7 (1.0) | 0 (0.0) | 1 (1.0) |
| Pulmonary embolism | 3 (0.3) | 4 (0.4) | 2 (0.3) | 4 (0.6) | 0 (0.0) | 0 (0.0) |

CV, cardiovascular; DD-CKD, dialysis-dependent chronic kidney disease; ESA, erythropoiesis-stimulating agent; HF, heart failure; MACE, major adverse cardiovascular events; MI, myocardial infarction; TE, thromboembolic event; VAT, vascular access thrombosis; wk, week.

**Table S2.** Change in hemoglobin from baseline for vadadustat treatment during primary and secondary evaluation periods by baseline ESA dose in patients with prevalent DD-CKD (randomized population)

|  | **Overall**  **n = 1777** | **≤90 U/kg/wk**  **n = 916** | **>90 and <300 U/kg/wk**  **n = 724** | **≥300 U/kg/wk**  **n = 102** |
| --- | --- | --- | --- | --- |
| **Weeks 24-36 (PEP)** | | | | |
| Baseline Hb concentration, g/dL,  mean (SD) | 10.25 (0.85) | 10.33 (0.81) | 10.22 (0.87) | 9.72 (0.91) |
| Hb concentration (observed + imputed), g/dL, mean (SD) | 10.36 (1.01) | 10.53 (0.99) | 10.24 (0.98) | 9.78 (0.95) |
| Change in Hb from baseline, LS mean  (95% CI) | 0.19 (0.12, 0.25) | 0.25 (0.17, 0.33) | 0.12 (0.02, 0.23) | –0.11 (–0.53, 0.30) |
| Difference in Hb change from darbepoetin alfa, LS mean (95% CI) | –0.17  (–0.23, –0.10) | –0.10  (–0.19, –0.02) | –0.20  (–0.30, –0.09) | –0.39  (–0.67, –0.11) |
| **Weeks 40-52 (SEP)** | | | | |
| Hb concentration (observed + imputed), g/dL, mean (SD) | 10.40 (1.04) | 10.60 (1.05) | 10.23 (0.99) | 9.81 (0.91) |
| Change in Hb from baseline, LS mean  (95% CI) | 0.23 (0.16, 0.29) | 0.33 (0.24, 0.41) | 0.10  (–0.01, 0.21) | 0.08  (–0.35, 0.51) |
| Difference in Hb change from darbepoetin alfa, LS mean (95% CI) | –0.18  (–0.25, –0.12) | –0.07  (–0.17, 0.02) | –0.27  (–0.37, –0.16) | –0.47  (–0.74, –0.20) |

DD-CKD, dialysis-dependent chronic kidney disease; ESA, erythropoiesis-stimulating agent; Hb, hemoglobin; LS, least-squares; PEP, primary evaluation period; SEP, secondary evaluation period; wk, week.

**Table S3.** Analysis of rescue therapy use by baseline ESA dose in patients with prevalent DD-CKD

| **Any ESA Rescue^a^** | | | | | | |
| --- | --- | --- | --- | --- | --- | --- |
|  | **≤90 U/kg/wk** | | **>90 and <300 U/kg/wk** | | **≥300 U/kg/wk** | |
|  | **Vadadustat (n = 913)** | **Darbepoetin Alfa  (n = 963)** | **Vadadustat (n = 720)** | **Darbepoetin Alfa  (n = 691)** | **Vadadustat (n = 102)** | **Darbepoetin Alfa  (n = 98)** |
| Weeks 2-8,^b^ n (%) | 57/913 (6.2) | 134/963 (13.9) | 84/720 (11.7) | 84/691 (12.2) | 27/102 (26.5) | 25/97 (25.8) |
| Weeks 10-20,  n (%) | 87/852 (10.2) | 139/935 (14.9) | 163/674 (24.2) | 109/668 (16.3) | 43/91 (47.3) | 28/94 (29.8) |
| Weeks 24-36  (PEP), n (%) | 78/768 (10.2) | 163/891 (18.3) | 138/600 (23.0) | 108/625 (17.3) | 32/77 (41.6) | 28/82 (34.1) |
| Weeks 40-52  (SEP), n (%) | 98/691 (14.2) | 131/830 (15.8) | 144/530 (27.2) | 104/566 (18.4) | 25/61 (41.0) | 23/77 (29.9) |
| Weeks 64-EOS,  n (%) | 105/527 (19.9) | 195/666 (29.3) | 122/368 (33.2) | 108/452 (23.9) | 23/48 (47.9) | 18/61 (29.5) |
| **RBC Transfusion** | | | | | | |
|  | **≤90 U/kg/wk** | | **>90 and <300 U/kg/wk** | | **≥300 U/kg/wk** | |
|  | **Vadadustat (n = 913)** | **Darbepoetin Alfa  (n = 963)** | **Vadadustat (n = 720)** | **Darbepoetin Alfa  (n = 691)** | **Vadadustat (n = 102)** | **Darbepoetin Alfa  (n = 98)** |
| Weeks 2-8,^b^ n (%) | 10/913 (1.1) | 5/963 (0.5) | 15/720 (2.1) | 6/691 (0.9) | 4/102 (3.9) | 3/97 (3.1) |
| Weeks 10-20,  n (%) | 16/852 (1.9) | 5/935 (0.5) | 17/674 (2.5) | 19/668 (2.8) | 5/91 (5.5) | 7/94 (7.4) |
| Weeks 24-36  (PEP), n (%) | 11/768 (1.4) | 6/891 (0.7) | 13/600 (2.2) | 14/625 (2.2) | 5/77 (6.5) | 1/82 (1.2) |
| Weeks 40-52  (SEP), n (%) | 8/691 (1.2) | 12/830 (1.4) | 16/530 (3.0) | 16/566 (2.8) | 1/61 (1.6) | 0/77 (0.0) |
| Week 64-EOS,  n (%) | 13/527 (2.5) | 16/666 (2.4) | 17/368 (4.6) | 18/452 (4.0) | 4/48 (8.3) | 3/61 (4.9) |

^a^In the darbepoetin alfa group, ESA was considered rescue medication if the dose was at least double that of the previous dose (this was defined post hoc). ^b^Starting at week 6, patients in both treatment groups could receive ESAs as rescue therapy if experiencing worsening symptoms of anemia of CKD with Hb <9.5 g/dL. Receiving rescue therapy between weeks 2 and 6 was a protocol violation.
DD-CKD, dialysis-dependent chronic kidney disease; EOS, end of study; ESA, erythropoiesis-stimulating agent; Hb, hemoglobin; PEP, primary evaluation period; RBC, red blood cell; SEP; secondary evaluation period; wk, week.

**Table S4.** Changes in median values of ferritin and C-reactive protein by baseline ESA dose during weeks 24-36 and 40-52 in patients with prevalent DD-CKD (randomized population)

| **Parameter**  **(Reference Range)** | **Baseline** | | **Weeks 24-36 (PEP)** | | **Weeks 40-52 (SEP)** | |
| --- | --- | --- | --- | --- | --- | --- |
|  | **Vadadustat,**  **Median**  **(Q1, Q3)** | **Darbepoetin Alfa,**  **Median**  **(Q1, Q3)** | **Vadadustat,**  **∆Median From Baseline  (Q1, Q3)** | **Darbepoetin Alfa,**  **∆Median From Baseline  (Q1, Q3)** | **Vadadustat,**  **∆Median From Baseline  (Q1, Q3)** | **Darbepoetin Alfa,**  **∆Median From Baseline  (Q1, Q3)** |
| **Baseline ESA dose subgroup: ≤90 U/kg/wk** | | | | | | |
| Ferritin, ng/mL  (10-380 ng/mL)^a^ | n = 916  797.0  (426, 1183) | n = 968  771.0  (439, 1184) | n = 804  –73.3  (–246.8, 110.0) | n = 875  –35.0  (–200.0, 176.0) | n = 748  –81.0  (–286.8, 112.2) | n = 827  –55.5  (–251.5, 177.0) |
| CRP, mg/L  (0.0-4.9 mg/L)^a^ | n = 904  4.0 (2.0, 10.0) | n = 947  4.0 (2.0, 10.0) | n = 525  0.0 (–2.0, 2.0) | n = 552  0.0 (–2.0, 4.0) | n = 638  0.0 (–2.0, 3.0) | n = 700  0.0 (–2.0, 4.0) |
| **Baseline ESA dose subgroup: >90 and <300 U/kg/wk** | | | | | | |
| Ferritin, ng/mL  (10-380 ng/mL)^a^ | n = 723  736  (397, 1135) | n = 693  721  (414, 1126) | n = 628  –22.6  (–235.9, 194.0) | n = 618  –12.3  (–208.0, 197.5) | n = 564  –23.8  (–255.5, 203.0) | n = 567  1.0  (–221.7, 213.5) |
| CRP, mg/L  (0.0-4.9 mg/L)^a^ | n = 716  4.5 (2.0, 11.0) | n = 680  4.0 (2.0, 10.0) | n = 403  0.0 (–2.0, 3.0) | n = 395  0.0 (–2.0, 3.0) | n = 477  0.0 (–2.0, 3.0) | n = 475  0.0 (–2.0, 3.0) |
| **Baseline ESA dose subgroup: ≥300 U/kg/wk** | | | | | | |
| Ferritin, ng/mL  (10-380 ng/mL)^a^ | n = 102  701  (415, 1019) | n = 98  748  (426, 1172) | n = 79  9.8  (–204.0, 221.0) | n = 83  –8.5  (–223.0, 175.5) | n = 76  57.8  (–166.8, 326.8) | n = 80  13.0  (–126.5, 228.5) |
| CRP, mg/L  (0.0-4.9 mg/L)^a^ | n = 102  4.0 (1.0, 15.0) | n = 96  5.0 (2.0, 12.0) | n = 49  0.0 (–4.0, 0.0) | n = 53  0.0 (–5.0, 4.0) | n = 61  0.0 (–2.0, 4.0) | n = 67  0.0 (–3.0, 3.0) |

^a^Population reference ranges.^1,2^
∆, change (from baseline); CRP, C-reactive protein; DD-CKD, dialysis-dependent chronic kidney disease; ESA, erythropoiesis-stimulating agent; PEP, primary evaluation period; Q1, lower quartile; Q3, upper quartile; SEP, secondary evaluation period; wk, week.

**References**

1. M. J. Koury, R. Agarwal, G. M. Chertow, et al., “Erythropoietic Effects of Vadadustat in Patients With Anemia Associated With Chronic Kidney Disease,” *American Journal of Hematology* 97, no. 9 (2022): 1178–1188.
2. A. Wu, *Tietz Clinical Guide to Laboratory Tests*, 4th ed. (Saunders/Elsevier, 2006).
